# Diagnosis of subjects damaged in Buddhist groups by means of the Symptom Checklist (SCL-90)

**DOI:** 10.1192/j.eurpsy.2022.1917

**Published:** 2022-09-01

**Authors:** A.I.M. Anders

**Affiliations:** Ludwig-Maximilians-University Munich, Institute Of Social And Cultural Anthropology, München, Germany

**Keywords:** Buddhist organizations, depressivity, paranoid thinking, psychoticism, SCL-90, social insecurity

## Abstract

**Introduction:**

At present, the mental health of members in international Buddhist organizations is often damaged by decontextualized concepts and misleading meditation training. As the treatment of resulting mental diseases presents therapeutic challenges, currently diagnostic and related therapeutic considerations are crucial.

**Objectives:**

Since subjects predominantly reported having received several diagnoses, with depression, anxiety disorder, and post-traumatic stress disorder being the most frequently assigned, a diagnostic assessment device was employed for further differentiation.

**Methods:**

The questionnaire *SCL-90* was used to evaluate the nine dimensions: *interpersonal sensitivity*, *depression*, *anxiety*, *paranoid ideation*, *psychoticism*, *somatization*, *obsessive-compulsive disorder*, *hostility* and *phobic anxiety.*

**Results:**

In a pilot group of eight German-speaking subjects of different Buddhist groups the general psychological burden (*GSI*) was significantly elevated in six of them. However, the intensity of responses in precisely those two individuals in whom it was not increased was far below the norm (*PSDI*). Furthermore, seven of the subjects had an above-average number of symptoms indicating burden (*PST*). All of them showed a heightened level of interpersonal sensitivity and for most of the subjects anxiety, depression, paranoid ideation and psychoticism were above the mean value of the norm group.

**Conclusions:**

As for psychiatric treatment and psychotherapy, extended research with a larger group of such subjects and at the beginning of their treatment is crucial. Particularly, hypotheses on the causes of their social insecurity, depressivity, paranoid thinking as well as psychoticism based on the distorted concepts and neologisms these persons were exposed to (e.g. ‘*karma-purification *’) as well as their ways of ‘meditation-training’ seems to hold core relevance.

**Disclosure:**

This research was funded by the German Federal Ministry of Education and Research, funding reference number: 01UL1823X.

